# RETRACTED: Arslan et al. CAVVPM: Challenge-Based Authentication and Verification of Vehicle Platooning at Motorway. *Sensors* 2022, *22*, 7946

**DOI:** 10.3390/s26113383

**Published:** 2026-05-27

**Authors:** Muhammad Arslan, Muhammad Faran Majeed, Rana Abu Bakar, Jawad Khan, Shafiq Hussain, Youngmoon Lee, Faheem Khan

**Affiliations:** 1 Department of Computing Sciences, University of Sahiwal, Sahiwal 57000, Pakistan; 2 Department of Computer Science, Kohsar University Murree, Murree 47150, Pakistan; 3 Department of Computer Engineering, Faculty of Engineering, Chulalongkorn University, Bangkok 10330, Thailand; 4 Department of Robotics, Hanyang University, Ansan 15588, Republic of Korea; 5 Department of Computer Engineering, Gachon University, Seongnam-si 13120, Republic of Korea

The journal retracts the article, titled “CAVVPM: Challenge-Based Authentication and Verification of Vehicle Platooning at Motorway” [1], cited above. 

Following publication, concerns were brought to the attention of the Editorial Office regarding the usage of unusual phrases and an overlap between this publication [1] and a previously published article [2], produced by a different authorship group.

Adhering to our standard procedure, the Editorial Office and Editorial Board conducted an investigation that confirmed the presence of non-sensical phrases and a significant overlap between the two above-mentioned publications, without appropriate acknowledgment or citation. As a result, the Editorial Board and the authors have decided to retract this paper as per MDPI’s retraction policy.

This retraction was approved by the Editor-in-Chief of the *Sensors* journal.

The authors have been informed of this retraction. Muhammad Arslan, Rana Abu Bakar, Shafiq Hussain, Youngmoon Lee, and Faheem Khan have indicated their agreement with this decision. The other authors did not provide a comment on this decision.
